# The Role of Respiratory Syncytial Virus Infection in the Hospitalization and Mortality of Adults with Congestive Heart Failure in Spain, 2018–2022

**DOI:** 10.3390/v17040516

**Published:** 2025-04-01

**Authors:** Rosa María Gómez-García, Rodrigo Jiménez-García, Ana López-de-Andrés, Valentín Hernández-Barrera, Ana Jimenez-Sierra, Natividad Cuadrado-Corrales, José Javier Zamorano-León, David Carabantes-Alarcón, Andrés Bodas-Pinedo, Javier De-Miguel-Díez

**Affiliations:** 1 Respiratory Care Department, Hospital General Universitario Gregorio Marañón, Instituto de Investigación Sanitaria Gregorio Marañón (IiSGM), Universidad Complutense de Madrid, 28007 Madrid, Spain; rosamg07@ucm.es (R.M.G.-G.); javier.miguel@salud.madrid.org (J.D.-M.-D.); 2 Department of Public Health & Maternal and Child Health, Faculty of Medicine, Universidad Complutense de Madrid, 28040 Madrid, Spain; mariancu@ucm.es (N.C.-C.); josejzam@ucm.es (J.J.Z.-L.); dcaraban@ucm.es (D.C.-A.); abodas@ucm.es (A.B.-P.); 3 Department of Public Health & Maternal and Child Health, Faculty of Pharmacy, Universidad Complutense de Madrid, 28040 Madrid, Spain; anailo04@ucm.es; 4 Preventive Medicine and Public Health Teaching and Research Unit, Health Sciences Faculty, Rey Juan Carlos University, 28922 Alcorcón, Spain; valentin.hernandez@urjc.es; 5 Faculty of Medicine, Universidad San Pablo Ceu, 28668 Madrid, Spain; a.jimenez100@usp.ceu.es

**Keywords:** heart failure, respiratory syncytial virus, hospitalizations, mortality, costs, Spain

## Abstract

(1) Background: Heart failure (HF) is a growing health concern, with decompensations being a major cause of hospital admissions. Respiratory syncytial virus (RSV) infection can trigger HF exacerbations, increasing morbidity and mortality. This study analyzed hospitalizations for HF with RSV infection in Spain from 2018 to 2022 using data from the Spanish Hospital Discharge Database. (2) Methods: We included patients aged ≥40 years with a diagnosis of HF, selecting a matched control group without RSV infection based on the HF diagnostic code position, year of admission, sex, and age. (3) Results: Of 424,413 HF hospitalizations, 0.47% (1988) involved RSV infection. Hospitalizations for HF with RSV increased over time, except for a decline in 2020–2021. ICU admissions and hospital length of stay decreased, but in-hospital mortality and costs remained unchanged. Patients with RSV had fewer comorbidities than those without RSV but experienced a higher mortality, more frequent non-invasive ventilation, longer hospital stays, and greater costs. Factors linked to higher mortality included advanced age, myocardial infarction, SARS-CoV-2 coinfection, and oxygen therapy dependence. RSV infection was independently associated with increased in-hospital mortality. (4) Conclusions: These findings highlight the need for early RSV detection in HF patients to implement preventive measures and reduce severe disease outcomes.

## 1. Introduction

Heart failure (HF) is a major global health issue with an increasing prevalence. Its natural progression is characterized by acute decompensation episodes, which are the leading cause of hospitalization. Hospital admissions signify a turning point in the disease course, as they increase the risk of recurrent hospitalizations and are associated with higher mortality rates [1,2,3].

Respiratory syncytial virus (RSV) infection is also a significant cause of hospital admissions, exhibiting strong seasonal patterns. For a long time, RSV infection in adults was underestimated, as it was primarily considered a severe disease in infants and young children [4]. However, the widespread use of rapid polymerase chain reaction (PCR) testing has improved the identification of the RSV burden across different populations. Severe cases predominantly affect children under 2 years, older adults, immunocompromised individuals, and those with underlying comorbidities [4,5,6,7].

RSV transmission occurs through mucosal contact with infected secretions via respiratory droplets, aerosolized particles, or contaminated surfaces. Hospital staff may facilitate nosocomial infections, as the virus remains viable for up to 5 h on gloves, 6 h on non-porous surfaces, and approximately 30 min on skin [8]. High-risk groups for severe RSV infection include children under 2 years, the elderly, immunosuppressed individuals, and patients with respiratory or cardiovascular comorbidities [9]. Viral shedding begins approximately 24 h after infection but varies depending on host age, disease severity, and immune status. Adults typically shed the virus for 3–7 days, whereas immunocompromised individuals may continue shedding for several months [8]. Immunosenescence plays a critical role in the development and severity of RSV infection [9].

RSV infection is associated with an increased mortality in vulnerable populations. A study conducted in Southern California (2011–2015) found that hospitalized adults over 60 years old had comparable in-hospital mortality rates between RSV and influenza infections. Notably, 35% of RSV-infected patients had HF [10].

Infections, particularly respiratory infections, are among the most common comorbidities in hospitalized HF patients. Respiratory infections trigger and exacerbate cardiac events through acute systemic inflammation, contributing to arrhythmias, myocardial ischemia, hypoxemia, and sympathetic activation, ultimately leading to HF decompensation [11]. RSV-associated respiratory disease is complicated by cardiovascular events in 14–22% of hospitalized adults, including worsening congestive HF, acute coronary syndrome, and arrhythmias. Additionally, underlying cardiovascular disease is present in 45–63% of hospitalized RSV cases [12]. A U.S. study found RSV hospitalization rates to be 4.0–33.2 times higher in HF patients compared to those without HF [13].

Discrepancies exist regarding the severity of RSV infection in HF patients. Falsey et al. did not observe an increased severity in HF patients [14], while other studies reported hospitalization rates eight times higher in HF patients compared to non-HF adults [15].

Understanding the epidemiology of RSV infection in high-risk populations, such as HF patients, is crucial for developing preventive strategies and guiding public health policies [16]. In recent years, RSV vaccines have been developed. In Spain, an available vaccine demonstrated a 73.8% efficacy against severe disease over two seasons in individuals with at least one cardiorespiratory comorbidity [17]. It is anticipated that vaccination will lead to a significant epidemiological shift, reducing morbidity and mortality, similar to what has been observed with SARS-CoV-2.

In this study, we aimed to describe and analyze the hospitalizations of patients with HF and RSV infection in Spain between 2018 and 2022. We also compared patients infected with RSV with those not infected with RSV to assess differences in hospital outcomes.

## 2. Materials and Methods

An epidemiological retrospective observational study was conducted with a descriptive approach. The data source was the Spanish Hospital Discharge Database (SHDD), which compiles demographic details (age and sex), primary and secondary diagnoses (up to 19 additional conditions), records of diagnostic and therapeutic interventions (a maximum of 20 per patient), ICU admissions, LOHS, discharge outcomes (recovery or death), and healthcare costs. Diagnoses and procedures in the SHDD are coded using the ICD-10 system, and further details are available online [18]. The study analyzed data from 1 January 2018 to 31 December 2022.

### 2.1. Participants

The population included patients hospitalized in Spain’s public hospitals from 2018 to 2022, with a diagnosis of congestive heart failure appearing in any of the 20 fields in the SHDD. Individuals under 40 years of age were excluded due to the low prevalence of congestive heart failure in this age group [19]. Cases were also excluded if key variables such as age, sex, admission and discharge dates, or discharge status were missing. Congestive heart failure patients were classified based on the presence or absence of an RSV infection identified through an ICD-10 code.

For comparability, each congestive heart failure patient with an RSV infection was matched with a congestive heart failure patient without an RSV infection. The matching was based on the position of the congestive heart failure code in diagnostic fields (1–20), the year of admission, age, and sex. If multiple matches met the criteria, one was selected randomly.

### 2.2. Study Variables

The main outcome was in IHM, calculated as the percentage of patients who died during hospitalization. Secondary outcomes included ICU admissions, use of mechanical ventilation (both invasive and non-invasive), dependence on supplemental oxygen, LOHS, and hospitalization costs. Other variables examined were patient demographics and comorbidities.

Comorbidities were assessed using the Charlson Comorbidity Index (CCI), adapted for administrative databases coded with ICD-10 [20,21]. Additional conditions, such as arterial hypertension, dyslipidemia, atrial fibrillation, asthma, chronic obstructive pulmonary disease (COPD), emphysema, bronchiectasis, acute bronchitis, bronchiolitis, influenza, COVID-19, obesity, pneumonia, depression, and obstructive sleep apnea (OSA), were also analyzed. The procedures studied included mechanical ventilation, long-term steroid use, and dependence on supplemental oxygen. ICD-10 codes for all variables are detailed in Table S1.

### 2.3. Statistical Analysis

We estimated the standardized incidence rate per 100,000 inhabitants per year and analyzed the time trend using Joint Point Regression (Joint Point Regression program, National Cancer Institute 2025).

Categorical variables were described as frequencies and percentages, while continuous variables were presented as means with standard deviations or medians with first and third quartile (Q1 and Q3) for those variables not normally distributed according to the Kolmogórov–Smirnov test (CCI, LOHS, and costs). Changes in variable distribution over the 2018–2022 period were analyzed using the Cochran–Armitage or Cochran–Mantel–Haenszel tests for categorical data and linear regression or Jonckheere–Terpstra tests for continuous data.

Group comparisons employed Fisher’s exact test for categorical variables and *t*-tests or Mann–Whitney tests for continuous variables.

Multiple testing correction was performed using the Benjamini and Hochberg method to control the false discovery rate. The procedure involved sorting the *p*-values from smallest to largest and setting the false discovery rate (q-value) as the proportion of *p*-values less than or equal to each value in the sorted list, divided by the position of the value in the list. Results with a q-value < 0.05 were considered significant [22].

To assess factors influencing IHM (dependent variable), multivariable logistic regression models were developed. Separate models were created for congestive heart failure patients with RSV, the matched congestive heart failure patients without RSV, and the overall group to evaluate RSV’s impact on mortality while controlling for confounders. In the logistic model of all patients, we included those heart failure patients with RSV and their matched control patients with congestive heart failure patients without RSV infection (n = 3976 patients).

The models were constructed according to the following steps. First, a bivariate analysis was conducted to assess the association between each independent variable and the dependent variables, identifying potential candidates for the multivariable model. All independent variables with a significant bivariate association (*p* < 0.1) and those considered scientifically relevant, including potential confounders based on the reviewed literature, were included in the initial model. For all models, we included the following variables: sex, age, dependence on supplemental oxygen, non-invasive mechanical ventilation, myocardial infarction, chronic renal disease, cancer, COPD, and COVID-19. RVS infection was only in the last model. Second, the importance of each independent variable was assessed using the Wald statistic, and variables that did not contribute significantly were eliminated through an iterative process, comparing models with the Likelihood Ratio test. Third, collinearity was evaluated using the Variance Inflation Factor (VIF), and potential two-way interactions were tested. Fourth, model fit was assessed using the Hosmer–Lemeshow test. Finally, odds ratios (ORs) with their corresponding 95% confidence intervals (CIs) were reported for each category of covariates independently associated with IHM.

All analyses were conducted using Stata version 14, with a *p*-value < 0.05 considered significant.

### 2.4. Ethical Considerations

The SHDD is managed by the Spanish Ministry of Health (SMH), which provides anonymized data to researchers upon application. Since the database is administrative and anonymized, Spanish law does not require ethics committee approval or informed consent for its use in epidemiological studies [18,23].

## 3. Results

Between 2018 and 2022, a total of 424,413 individuals aged 40 years or older with a diagnosis of congestive heart failure were admitted to public hospitals in Spain. Of these patients, 1988 (0.47%) were identified as having a recorded diagnosis of RSV infection.

### 3.1. Temporal Trends in Congestive Heart Failure and RSV-Related Hospitalizations and Associated Characteristics

As outlined in Table 1, the annual number of hospitalizations for congestive heart failure with RSV infection rose between 2018 and 2019, decreased slightly in 2020, experienced a sharp decline in 2021, and subsequently rebounded in 2022. The estimated standardized incidence rate per 100,000 inhabitants ranged from 0.40 in the year 2021 to 2.52 in the year 2020, with no significant variation overtime according to Joint Point Regression (*p* = 0.857).

The proportion of congestive heart failure patients hospitalized with an RSV infection increased significantly from 0.37% in 2018 to 0.75% in 2022 (*p* < 0.001). The distribution between men and women was nearly equal (50.6% vs. 49.4%; *p* = 0.425). The mean age remained stable at approximately 80 years throughout the study period, as did the median CCI (*p* = 0.609).

The proportion of patients requiring ICU admission steadily declined, from 6.11% in 2018 to 2.87% in 2022 (*p* = 0.002). IHM exhibited a modest increase over the study period, though no statistically significant trend was observed (9% in 2018 vs. 9.77% in 2022), with a marked peak in 2020 (13.64%). The median LOHS decreased significantly from 9 days in 2018 to 7 days in 2022. Hospitalization costs remained stable between 2018 and 2022.

### 3.2. Characteristics, Outcomes, and Comorbidities of Congestive Heart Failure Patients Hospitalized with and Without RSV Infection

Table 2 provides a comparative analysis of hospitalizations involving congestive heart failure patients with RSV infection and matched congestive heart failure patients without RSV infection based on year of admission, age, and sex. Following matching, the median CCI was significantly higher in the non-RSV group (3 versus 4; *p* < 0.001). Conversely, patients with ab RSV infection demonstrated higher rates of non-invasive mechanical ventilation (9.46% versus 4.53%; *p* < 0.001), IHM (10.16% versus 8.25%; *p* = 0.037), LOHS (8 versus 7 days; *p* < 0.001), and median hospitalization costs (3740 euros vs. 3616, *p* < 0.001).

When comparing comorbidities, as shown in Table 3, the most prevalent chronic conditions included COPD (66.5% in patients with RSV versus 71.08% in patients without RSV; *p* = 0.006), atrial fibrillation (51.51% versus 52.01%; *p* = 0.908), and dyslipidemia (39.39% versus 41.85%; *p* = 0.179). Asthma was significantly more frequent in patients with an RSV infection (29.93% versus 24.85%; *p* < 0.001), as were acute bronchitis (17.15% versus 3.57%; *p* < 0.001) and other respiratory conditions such as influenza and bronchiolitis. Conversely, diagnoses of myocardial infarction (*p* = 0.008), peripheral vascular disease (*p* < 0.001), cerebrovascular disease (*p* = 0.010), cancer (*p* = 0.004), COPD (*p* = 0.006), and COVID-19 (*p* < 0.001) were less frequent in patients with an RSV infection.

### 3.3. Multivariable Analysis of Factors Associated with IHM in Congestive Heart Failure Patients with and Without RSV Infection

The variables linked to IHM are presented in Table 4. Factors associated with a higher IHM in both congestive heart failure patients with and without an RSV infection included an older age, myocardial infarction, COVID-19, and dependence on supplemental oxygen. Chronic renal disease and cancer were factors associated with IHM in patients without an RSV infection. Concomitant COPD was associated with IHM only among patients with an RSV infection.

The use of non-invasive mechanical ventilation was associated with a higher IHM in patients with an RSV infection (OR 3.32; 95%CI 2.17–5.09).

In the overall study population, the presence of an RSV infection was associated with a modest but statistically significant increase in IHM (OR 1.29; 95% CI 1.01–1.59) after multivariable adjustment.

## 4. Discussion

We found that only 0.47% of hospitalized HF patients had an RSV infection. Moreover, we observed an increase in cases between 2018 and 2019, followed by a decline in 2020, a further drop in 2021, and a subsequent resurgence in 2022. The reductions in prevalence can be attributed to COVID-19 containment measures during 2020–2021, including social isolation, movement restrictions, mask usage, and physical distancing. Conversely, the later increase may be related to the relaxation of these measures and the expanded use of PCR testing, which allows for better virus detection [24,25].

Over time, the profile of HF patients with an RSV infection remained unchanged, with a similar sex distribution, an average age of approximately 80 years, and a stable Charlson Comorbidity Index. Although literature on this topic is scarce, it is known that HF patients with an RSV infection are more likely to develop severe disease. Landi et al. examined the hospitalizations of patients diagnosed with an RSV infection in the U.S. over six seasons (2016–2022), finding a higher hospitalization rate among high-risk patients, particularly those over 65 years or with comorbidities such as COPD, HF, or asthma [26]. However, cross-country comparisons remain challenging. Savarese et al., in their global review of HF epidemiology, highlighted a significant heterogeneity among populations, with notable geographic variations. HF prevalence is rising due to aging populations, advances in treatments, and the availability of more effective therapies, but these factors differ across countries [27].

Despite the increased severity of RSV-associated HF, our study showed a decrease in ICU admissions over time, from 6.11% in 2018 to 2.87% in 2022. In the general RSV-infected population, reported ICU admission rates were higher. Tseng et al. described a 17.9% ICU admission rate in RSV-infected adults over 60 years in California [28]. Similarly, Havers et al. found an ICU admission rate of 19.1% among RSV-infected adults over 18 years hospitalized in the U.S. between 2016 and 2023 [29]. Additionally, Alfano et al. conducted a literature review reporting that 10–13% of RSV-hospitalized adults required ICU admission, attributing severe disease to multifactorial causes, including immunosenescence, hypoxia, and febrile syndrome [30]. Differences between studies may be due to variations in their study populations influenced by age, race, and geographic origin.

During the study period, we also observed a decrease in LOHS, from 9 days in 2018 to 7 days in 2022. The reported LOHS among RSV-hospitalized patients varies widely. Havers et al. reported a shorter LOHS (4.1 days) among U.S. adults ≥60 years hospitalized between October 2022 and April 2023 [31]. This variability may be explained by differences in age, race, healthcare systems, and study settings across countries.

IHM remained stable throughout the study, except during the COVID-19 pandemic, when it increased. There are no specific series measuring mortality in HF patients with an RSV infection, but studies have analyzed mortality in RSV-infected adults. Tseng et al. reported a lower IHM (5.6%) in RSV-hospitalized adults over 60 years (2011–2015) [28], as did Havers et al. (4.7%) in U.S. adults over 60 years (2016–2023) [29]. However, other studies reported higher mortality rates, ranging from 11% to 18%, in RSV-infected patients over 65 years [24,30,32,33]. The differences in mortality may be attributed to our study’s inclusion of adults over 40 years, potentially resulting in a lower mortality since HF alone does not explain the reduced mortality risk. Additionally, variations between studies may arise from differences in methodologies, inclusion criteria, case definitions, study populations, RSV epidemiology, and healthcare-seeking behaviors [32].

When comparing HF patients with and without an RSV infection, we found that the median CCI was significantly higher in the non-RSV group (4 vs. 3; *p* < 0.001). Despite having fewer comorbidities, the RSV-infected group exhibited greater severity, with higher rates of non-invasive mechanical ventilation (NIMV), IHM, and LOHS, leading to higher hospitalization costs. While extensive literature exists on HF-related costs, no studies specifically compare the costs associated with RSV infection. Kwok et al. analyzed HF-related costs in U.S. inpatients (2010–2014), finding mean total costs of USD 15,618 ± USD 25,264 for patients readmitted within 30 days and USD 11,845 ± USD 22,710 for those without readmission. They identified factors associated with higher hospital costs, including invasive procedures (e.g., coronary angiography, mechanical ventilation) and comorbidities such as cardiopulmonary disorders, valvular heart disease, and bleeding [34]. Wyffels et al. analyzed U.S. adults diagnosed with RSV (2011–2015), finding that high-risk patients (with COPD, HF, or immunosuppression) had an additional cost burden of USD 9210 per patient after an RSV diagnosis [35]. Carrico et al. estimated an annual RSV burden of 4.0 million cases and USD 6.6 billion in economic costs in U.S. adults over 60 years, with the highest impact among HF patients [36].

The most common comorbidities in both HF groups (with and without RSV) were COPD, atrial fibrillation, and dyslipidemia. HF patients with an RSV infection had a higher prevalence of asthma and were more frequently diagnosed with acute bronchitis, bronchiolitis, or influenza coinfection. In contrast, HF patients without an RSV infection had higher rates of cardiovascular, respiratory, and oncological comorbidities. Risk factors for increased mortality included an advanced age, myocardial infarction, SARS-CoV-2 coinfection, and oxygen therapy dependence. In the RSV-infected group, COPD was associated with a higher IHM. Similarly, Carballo et al. found increased mortality in COPD patients [37]. Several studies report a 10–40% prevalence of HF-COPD coexistence due to shared pathogenic mechanisms, and this association is linked to an increased disease severity [38,39].

One of the most notable findings of our study was the higher IHM in RSV-infected patients after multivariable adjustment (OR 1.29; 95% CI 1.01–1.59). Similarly, Ivey et al. found higher RSV-associated mortality rates in their 2018 review [12]. Falsey et al. also reported an increased mortality in RSV-infected patients compared to those with cardiac conditions without RSV [40]. Hansen et al. analyzed U.S. death certificates (1999–2018), finding higher RSV mortality rates in adults over 65 years, with a stable annual mortality but geographic variations [25]. Tseng et al. studied RSV-hospitalized adults over 60 years (2011–2015) and reported an IHM of 5.6%, with higher mortality in those over 75 years. They identified advanced age, prior hospitalizations, and HF decompensation as predictors of medium- and long-term mortality [28].

In our study, it is striking that cancer and chronic kidney disease are not factors associated with hospital mortality among HF hospitalized patients with an RSV infection. Although the cause is unknown, it is possible that these patients are admitted with a less serious disease when they have an RSV infection because of the clinicians’ fear that they will show a poor outcome. In the same line, Cole et al. also described a low mortality associated with RSV infection in pediatric oncology patients [41].

The strengths of our study include the large sample size, five-year consecutive data analysis, and the use of the SNHDD. However, our study has limitations. The data source is administrative rather than clinical, meaning diagnostic codes do not capture disease severity or causal relationships. The registry is heterogeneous and lacks a standardized collection protocol, as diagnoses originate from hospital discharge reports across multiple centers and physicians. On the other hand, patients with more severe baseline conditions are more likely to be diagnosed with RSV, potentially leading to an overestimation of ICU admissions and mortality rates. Furthermore, the SHDD does not include data on pharmacological therapy. Another limitation is the racial homogeneity of the Spanish population, which may limit the generalizability of our findings to other regions.

The number of variables included in the multivariable models may seem high. However, the rule of thumb classically says that logistic models should be used with a minimum of 10 outcome events per predictor variable [42]. We constructed three models, for patients with RSV (outcome variable N = 202), no RSV (outcome variable N = 164), and all patients (outcome variable N = 366). The number of predictor variables for the three models were 11, 11, and 12 respectively. Finally, we were unable to analyze the effect of RSV vaccination in patients with HF, as these vaccines were approved for use in Spain after the period evaluated in this study.

## 5. Conclusions

In conclusion, the prevalence of RSV infection in HF patients increased throughout the study period. Despite having a lower comorbidity burden, HF patients with an RSV infection exhibited a greater disease severity, including longer hospital stays, a higher mortality, and consequently, increased hospital costs. Therefore, the early detection of RSV infection is crucial to implementing measures that prevent complications and reduce the mortality associated with severe disease. The use of vaccination and other preventive strategies could help mitigate RSV-related complications and mortality in patients with heart failure.

## Figures and Tables

**Table 1:** Characteristics of patients hospitalized with a diagnosis of congestive heart failure and respiratory syncytial virus infection in Spain (2018–2022).

Characteristics		2018	2019	2020	2021	2022	*p* Trend
Number of patients		311	481	390	110	696	
Standardized incidence rate. Per 100,000 inhabitants *		1.19	1.81	1.44	0.40	2.52	0.857
Sex n (%)	Men	167 (53.7)	251 (52.18)	200 (51.28)	51 (46.36)	337 (48.42)	0.425
	Women	144 (46.3)	230 (47.82)	190 (48.72)	59 (53.64)	359 (51.58)	
Age, mean (SD)		80,13 (8.61)	80.74 (8.73)	80.61 (9.08)	82.59 (9.94)	81.48 (9.48)	0.056
Age groups, n (%)	40–64 years	17 (5.47)	22 (4.57)	19 (4.87)	8 (7.27)	42 (6.03)	0.629
	64–74 years	57 (18.33)	93 (19.33)	69 (17.69)	14 (12.73)	109 (15.66)	
	75+ years	237 (76.21)	366 (76.09)	302 (77.44)	88 (80)	545 (78.3)	
CCI, n (%)	0	28 (9)	50 (10.4)	40 (10.26)	15 (13.64)	91 (13.07)	0.424
	1–2	96 (30.87)	163 (33.89)	128 (32.82)	41 (37.27)	222 (31.9)	
	3+	187 (60.13)	268 (55.72)	222 (56.92)	54 (49.09)	383 (55,03)	
CCI, edian (Q1–Q3)		3 (2–4)	3 (2–5)	3 (2–4)	4 (2–5)	3 (2–5)	0.909
Admission to ICU n (%)		19 (6.11)	34 (7.07)	29 (7.44)	3 (2.73)	20 (2.87)	0.002
IHM n (%)		28 (9)	51 (10.6)	40 (10.26)	15 (13.64)	68 (9.77)	0.711
LOHS, median (Q1–Q3)		9 (5–14)	9 (6–14)	8 (5–13)	8 (6–14)	7 (4.5–12)	0.011
Costs in euros, median (Q1–Q3)		3226 (3052–3660)	3302 (3289–4522)	4015 (3619–4964)	3878 (3428–4843)	3616 (3063–4975)	<0.001

* Time trend was analyzed with Joint Point Regression. CCI: Charlson Comorbidity Index. IHM: in-hospital mortality. LOHS: length of hospital stay. ICU: intensive care unit. SD: standard deviation. Q1–Q3: first and the third quartiles.

**Table 2:** Characteristics of patients hospitalized with a diagnosis of congestive heart failure and respiratory syncytial virus infection (RSV) and age–sex-matched controls without syncytial virus infection in Spain (2018–2022).

Characteristics		RSV	NO RSV	*p*
Sex n (%)	Men	1006 (50.6)	1006 (50.6)	NA
	Women	982 (49.4)	982 (49.4)	
Age, mean (SD)		80.98 (9.13)	80.98 (9.13)	NA
Age groups, n (%)	40–64 years	108 (5.43)	108 (5.43)	NA
	64–74 years	342 (17.2)	342 (17.2)	
	75+ years	1538 (77.36)	1538 (77.36)	
CCI, n (%)	0	224 (11.27)	172 (8.65)	<0.001
	1–2	650 (32.7)	564 (28.37)	
	3+	1114 (56.04)	1252 (62.98)	
CCI, median (Q1–Q3)		3 (5–2)	4 (5–2)	<0.001
Invasive mechanical ventilation, n (%)	Yes	45 (2.26)	36 (1.81)	0.312
Non-invasive mechanical ventilation n (%)	Yes	188 (9.46)	90 (4.53)	<0.001
Dependence on supplemental oxygen n (%)	Yes	296 (14.89)	330 (16.6)	0.139
Admission to ICU n (%)	Yes	105 (5.28)	104 (5.23)	0.943
IHM n (%)	Yes	202 (10.16)	164 (8.25)	0.037
LOHS, median (Q1–Q3)		8 (13–5)	7 (12–4)	<0.001
Costs in euros, median (Q1–Q3)		3616 (4522–3199)	3740 (5138–3226)	<0.001

CCI: Charlson Comorbidity Index. IHM: in-hospital mortality. LOHS: length of hospital stay. NA: not available. Q1–Q3: first and the third quartiles.

**Table 3:** Comorbidities and coinfections of patients hospitalized with a diagnosis of congestive heart failure and respiratory syncytial virus infection (RSV) and age–sex-matched controls without syncytial virus infection in Spain (2018–2022).

Comorbidities and Coinfections	RSV	NO RSV	*p*	q-Value
Arterial hypertension, n (%)	252 (12.68)	281 (14.13)	0.177	0.260
Dyslipidemia, n (%)	783 (39.39)	832 (41.85)	0.114	0.179
Atrial fibrillation, n (%)	1024 (51.51)	1034 (52.01)	0.751	0.908
Myocardial infarction, n (%)	22 (1.11)	46 (2.31)	0.003	0.008
Chronic renal disease, n (%)	663 (33.35)	715 (35.97)	0.083	0.152
Depression, n (%)	88 (4.43)	87 (4.38)	0.938	0.938
Diabetes, n (%)	731 (36.77)	736 (37.02)	0.869	0.910
Liver disease, n (%)	98 (4.93)	95 (4.78)	0.825	0.908
Peripheral vascular disease, n (%)	139 (6.99)	206 (10.36)	<0.001	<0.001
Cerebrovascular disease, n (%)	86 (4.33)	127 (6.39)	0.004	0.010
Cancer, n (%)	113 (5.68)	165 (8.3)	0.001	0.004
Asthma, n (%)	595 (29.93)	494 (24.85)	<0.001	<0.001
COPD, n (%)	1322 (66.5)	1413 (71.08)	0.002	0.006
Emphysema, n (%)	132 (6.64)	148 (7.44)	0.321	0.441
Bronchiectasis, n (%)	192 (9.66)	197 (9.91)	0.790	0.908
Acute bronchitis, n (%)	341 (17.15)	71 (3.57)	<0.001	<0.001
Bronchiolitis, n (%)	37 (1.86)	3 (0.15)	<0.001	<0.001
Influenza, n (%)	58 (2.92)	36 (1.81)	0.022	0.048
COVID-19, n (%)	28 (1.41)	109 (5.48)	<0.001	<0.001
Pneumonia, n (%)	168 (8.45)	205 (10.31)	0.044	0.088
Obesity, n (%)	414 (20.82)	373 (18.76)	0.103	0.174
OSA, n (%)	305 (15.34)	322 (16.2)	0.459	0.594

OSA: obstructive sleeping apnea. COPD: chronic obstructive pulmonary disease. Q-value test for multiple testing correction.

**Table 4:** Factors associated with in-hospital mortality among patients hospitalized with a diagnosis of congestive heart failure according to the presence of respiratory syncytial virus infection (RSV) in Spain (2018–2022).

Factors	RSV OR (95% CI)	No RSV OR (95% CI)	All Patients OR (95% CI)
40–64 years	1	1	1
65–74 years	1.71 (0.65–4.52)	1.63 (0.62–4.29)	1.62 (0.83–3.18)
75 years or more	3.53 (1.41–8.87)	3.45 (1.37–8.64)	3.41 (1.81–6.45)
Dependence on supplemental oxygen	1.53 (1.05–2.35)	1.44 (1.08–1.98)	1.45 (1.15–1.75)
Non-invasive mechanical ventilation	3.32 (2.17–5.09)	1.72 (0.95–3.13)	2.54 (1.81–3.56)
Myocardial infarction	5.77 (2.19–15.15)	1.95 (1.11–3.81)	2.58 (1.42–4.69)
Chronic renal disease	1.3 (0.94–1.81)	1.42 (1.05–1.92)	1.34 (1.08–1.67)
Cancer	0.98 (0.51–1.89)	2.83 (1.87–4.28)	2.03 (1.45–2.85)
COPD	1.46 (1.06–2.65)	1.06 (0.56–2.04)	1.14 (0.72–1.8)
COVID-19	4.19 (1.74–10.12)	2.37 (1.41–3.98)	2.55 (1.64–3.96)
Respiratory syncytial virus	NA	NA	1.29 (1.01–1.59)

COPD: chronic obstructive pulmonary disease. NA: not available.

## Data Availability

According to the contract signed with the Spanish Ministry of Health and Social Services, which provided access to databases from the Spanish National Hospital Database, we cannot share the databases with any other investigator, and we have to destroy the databases once the investigation has concluded. Consequently, we cannot upload the databases to any public repository. However, any investigator can apply for access to the databases by filling out the questionnaire available at https://www.sanidad.gob.es/estadEstudios/estadisticas/estadisticas/estMinisterio/SolicitudCMBD.htm (accessed on 26 October 2024). All other relevant data are included in the paper.

## Supplementary Materials

The following supporting information can be downloaded at: https://www.mdpi.com/article/10.3390/v17040516/s1, Table S1: International Classification of Diseases 10th Revision (ICD10) codes used in this investigation.

## Abbreviations

The following abbreviations are used in this manuscript:

CCI	Charlson Comorbidity Index
COPD	Chronic obstructive pulmonary disease
HF	Heart failure
ICU	Intensive care unit
IHM	In-hospital mortality
LOHS	Length of hospital stay
NIMV	Non-invasive mechanical ventilation
OSA	Obstructive sleep apnea
PCR	Polymerase chain reaction
RSV	Respiratory syncytial virus
SHDD	Spanish Hospital Discharge Database
SMH	Spanish Ministry of Health
